# Supplementary material for: Phage Encounters Recorded in CRISPR Arrays in the Genus Oenococcus
Source: Viruses. 2022 Dec 20;15(1):15. doi: 10.3390/v15010015 (PMC9867325; doi:10.3390/v15010015)
Supplement: Supplementary file 1 [file viruses-15-00015-s001.zip › viruses-2003343-supplementary.pdf]

viruses

MDPI

Table S1. Phage, bacterial genome and plasmid sequences used in the study.

Accession numbers of the 134 prophages earlier described [19]

Strain characteristics	Genome accession number
Lysogens (134)	ACSE01, AJIJ01, AJTO01, ALAD01, ALAE01, ALAG01, ALAH01, ALAJ01, ALAK01, AZHH01, AZIP01, AZJU01, AZJV01, AZJW01, AZJX01, AZJY01, AZKA01, AZKB01, AZKC01, AZKE01, AZKF01, AZKH01, AZKJ01, AZKL01, AZLG01, AZLK01, AZLN01, AZLP01, CP014324, CP027431, CP038451, JPEK01, JPEM01, LKRT01, LKRU01, LKRV01, LKRZ01, LKSA01, LKSB01, LKSC01, LKSD01, LKSE01, LKSR01, MLKP01, MLKQ01, MLKT01, MLKZ01, MLLA01, MLLB01, MLLD01, MLLE01, MLLG01, MLLK01, MLLM01, MLLN01, MLLO01, MLLQ01, MLLS01, MLLU01, MLLX01, MLLZ01, MLMF01, MLMI01, MLMJ01, MLMK01, MLMP01, MLMT01, MLMW01, MLMY01, MLMZ01, MLNA01, MLNC01, MLND01, MLNE01, MLNH01, MLNK01, MLNL01, MLNN01, MLNP01, MLNQ01, MLNS01, MLNU01, MLNV01, MLNX01, MLNY01, MLNZ01, MLOA01, MLOB01, MLOC01, MLOE01, MLOF01, MLOG01, MLOH01, MLOI01, MLOJ01, MLOL01, MLON01, MLOO01, MLOP01, MLOU01, MLOW01, MLOX01, MLOY01, MLPB01, MLPC01, MLPD01, MLPE01, MLPG01, MLPH01, MLPI01, MLPK01, MLPL01, MLPN01, MLPQ01, MLPR01, MLPS01, MLPT01, MLPV01, MLPW01, MLPX01, MLPY01, SNST01, SNSU01, SNSV01, SNSW01, SNSX01, SNSY01, ULFS01, ULFU01, ULFZ01, ULGA01, ULGB01, ULGC01, ULGD01.

Accession numbers of the selected temperate oenoprophages used for interspecies CRISPR targeting

Prophages	Lysogenic strains and GenBank assembly Accession		Reference
O. oeni			
AWRIB508 $_{\text{proA}}$	*O. oeni* AWRIB508	GCA_001869485.1	19
CRBO14223 $_{\text{proA}}$	*O. oeni* CRBO14223	OP964055	This study
AWRIB422 $_{\text{proA}}$	*O. oeni* AWRIB422	NZ_ALAG00000000	
IOEB1491 $_{\text{proA}}$	*O. oeni* IOEB1491	GCA_000762065.1	
IOEBS161 $_{\text{proA}}$	*O. oeni* IOEBS161	GCA_000762245.1	
IOEBB10 $_{\text{proB}}$	*O. oeni* IOEBB10	GCA_000761865.1	
AWRIB304 $_{\text{proC}}$	*O. oeni* AWRIB304	GCA_000286015.1	
IOEBL26-1 $_{\text{proB}}$	*O. oeni* IOEBL26-1	GCA_000762145.1	
AWRIB847 $_{\text{proB}}$	*O. oeni* AWRIB847	GCA_001869725.1	
AWRIB133 $_{\text{proB}}$	*O. oeni* AWRIB133	GCA_001939345.1	
AWRIB202 $_{\text{proB}}$	*O. oeni* AWRIB202	GCA_000309425.1	19
AWRIB150 $_{\text{proB}}$	*O. oeni* AWRIB150	GCA_001868205.1	
CRBO11105 $_{\text{proB}}$	*O. oeni* CRBO11105	GCA_002462335.1	
IOEB0501 $_{\text{proA}}$	*O. oeni* IOEB0501	GCA_000721875.1	
CRBO14221 $_{\text{proB}}$	*O. oeni* CRBO14221	OP964054	This study
IOEB0205 $_{\text{proA}}$	*O. oeni* IOEB0205	GCA_000721835.1	
CRBO14203 $_{\text{proA}}$	*O. oeni* CRBO14203	GCA_002462565.1	
AWRIB127 $_{\text{proC}}$	*O. oeni* AWRIB127	GCA_001868115.1	
AWRIB424 $_{\text{proB}}$	*O. oeni* AWRIB424	GCA_001868555.1	
CRBO14210 $_{\text{proB}}$	*O. oeni* CRBO14210	GCA_002462395.1	
IOEB0608 $_{\text{proA}}$	*O. oeni* IOEB0608	GCA_000761585.1	
UBOCC315001 $_{\text{proF}}$	*O. oeni* UBOCC315001	GCA_900518825.1	
IOEBS28 $_{\text{proC}}$	*O. oeni* IOEBS28	GCA_000761845.1	
CRBO1384 $_{\text{proE}}$	*O. oeni* CRBO1384	GCA_900518765.1	
IOEB0502 $_{\text{proA}}$	*O. oeni* IOEB0502	GCA_000761575.1	
IOEB9805 $_{\text{proD}}$	*O. oeni* IOEB9805	GCA_000761725.1	19
IOEBS13 $_{\text{proD}}$	*O. oeni* IOEBS13	GCA_000761785.1	
AWRIB418 $_{\text{proD}}$	*O. oeni* AWRIB418	GCA_000286155.1	
AWRIB663 $_{\text{proE}}$	*O. oeni* AWRIB663	GCA_001869605.1	
O. sicerae			

45.3 kb prophage	UCMA15228^{T} (1291631-1337008 bp)	NZ_CP029684	This study

**Free phages**	**Accession number**	**Reference**
OE33PA	MH220877	18
Vinitor162	MF939898	21, 22
Vinitor 27	MT859305	21 22
Vinitor 23	OP964053	21, 22
OE33PA6	OP964052	This study

**Other bacterial genomes strains**		
L. lactiplantibacillus plantarum QHM38203	CP028334.1	none
Liq. mali LM596	NZ_CP045035.1	none
O. alcoholitolerans UFRJ-M7.2.18^{T}	NR_134780	13
O. kitaharae DSM17330^{T}	NZ_AFVZ00000000	14
O. sicerae OAL24*	NZ_CP029684	16
O. kitaharae CRBO2176	NZ_JANJQP010000000	35

**Plasmids**	**Accession number**	**Reference**
pOeni 1 (*O. oeni*); 18.3 kb; theta-like replication	JX416328	58
pOeni 2 (*O. oeni*); 21.9 kb; theta-like replication	JX416329	58
pRSE2 (*O. oeni*); 2.544 kb; rolling-circle replication	NC_003201	59
pRSE3 (*O. oeni*) ; 3.948 kb; rolling-circle replication	NC_003099	59
pAWRIB429 (*O. oeni*)	NZ_CP084702	60

* the genome of *O. sicerae* OAL24 is a metagenome-assembled genome from water kefir.

Table S2. List of primers used in our study.

Target	Primers	Phage/lysogenic strain	Sequence	Reference
intA	IntA F	IOEB0608	CGAAGTTTTGACTGGAAAAGAAA	19, 24
	IntA R		TTGAGCGAAGCTGCTATAAGAAC	
intB	IntB F	IOEBB10	AGTTACCACCAAAGGCCATAAAC	
	IntB R		GCTCTGACGACTTACCAGCTTTA	
intC	IntC F	S28	GGCTATGACGCAGGGCGTGG	
	IntC R		TGACGGGACGTGCTGGCAAG	
intD	IntD F	IOEB9805	CGGAAAATATTATCAAGCACGAG	
	IntD R		TTCAGCGTGATCTTTACCAAAAT	
intE	intE F	CRBO1384	GATTTTTTGCCTGTAACGGTG	
	intE R		CGCAGGTATTCATGAGGATTC	
intF	intF/OC F	UBOCC31005	GATAGCAACACTGGAATGCC	
	intF/OC R		GCTATGGACGGTTATGGCAA	
tmp1	tmp1 F	IOEB B10	TGGAAGTTTGCTTAGTTGCAG	24
	tmp1 R		CAGCGGATGAAAACCATTGAGA	
tmp2	tmp2 F	IOEB L26	GGTTGGATCGGAAATTGCTA	
	tmp2 R		TGCAGCTATGGCTGTAATCG	
tmp3	tmp3 F	IOEB 0608	TGGTTCTGTTGTTCCTGGTATT	
	tmp3 R		GATTTAGAWGCCAGCTTTGCTA	
tmp4	tmp4 F	IOEB S28	CAACAAGCTGTTTTGGATCGC	
	tmp4 R		TAGCTTCTGCCATACTCTTAGC	
tmp5	tmp5 F	IOEB 9805	GGCCAAGGTACCGCAAAG	
	tmp5 R		CTCTGCTTTTCCAGTTTGCTG	
tmp6	tmp6cri F	Vinitor162	ATTCGCTCACAGGGGCAAAA	This study
	tmp6cri R		CCAAATTAGCAGTTGTCATTGTGCC	
tmp7	tmp7 F	UBOCC31005	TTGCAAGCTGAAGGTAAACAAT	
	tmp7 R		GCAGCTGTTTCATTGACTCTAA	
lm1	LM1_F	IOEB0608	AAAAGCATGATTTCCCGTTG	
	LM1_R		TGGCAATTCTATCAGGCACA	
lm2	LM2_F	IOEB0501	CAATGGATCAGCTTGCAGAA	
	LM2_R		GGTGCATCCATAGCAACTGT	
lm3	LM3_F	IOEB0502	GAAAATGCAGTTCCCGAAAG	
	LM3_R		CGCACGGTTTTATCAAGACA	
lm4	LM4_F	CRBO14203	TGATAAGATCCAAAGTGAAGCG	
	LM4_R		TCATCGGTCATATCAGGATCAG	
lm5	LM5_F	IOEBB10	CTGGTGTAATTGTGCTAATCGT	
	LM5_R		CCCGAATCAAGCAACTTCTTAA	
lm6	LM6_F	IOEB9805	AAGACCCAAAATCAGGCAATTT	
	LM6_R		AATTTGTTCATCAGTGGCACTT	
rep1	Rep1_F	IOEB0608	TGAACAGTGACGAAAAACGA	
	Rep1_R		TGCTTTCTCCTCTGTCTTTTCTT	
rep2	Rep2_F	OE33PA	ATTTGGATTGCCAGACGAAG	
	Rep2_R		CTTTCGCAAGCTTAGCGTCT	
rep3	Rep3_F	AWRIB418	ATTACCGATGAAACTTTGACCG	
	Rep3_R		TTCTAATTGCTTGACTCCCTGA	
rep4	Rep4_F	UBOCC31005	ACCAAAACTCAGTCAAGACAAC	
	Rep4_R		CTGGTGTGCTTCTTTACTTTCA	
rep5	Rep5_F	Vinitor 162	AGGCGATATCAAACAAGCAATT	
terS1	TerS1_F	L26-1	TGTCAATCCAGTCACGGGTA	
	TerS1_R		TGATCTGCTCGGCAGTAGAA	
terS2	TerS2_F	IOEB1491	ACACGCTGGAAGGAAAAGAA	
	TerS2_R		TCTTAATTCGGGCACCAAAG	
terS3	TerS3_F	IOEB0608	TTCAAGATTCCACCGGAAAG	

	TerS3_R		TCTTTCGGCTGAACGATTTT
terS4	TerS4_F	IOEB0502	GCCATGTGGAGAAAAATCGT
	TerS4_R		TAACCAGCAAAATCCGTTCC
terS5	TerS5_F	IOEB9805	AGACGGCGTAATAGTTGAGTTA
	TerS5_R		CATCTTGATCGCTTTCTTTCCA
terS6	TerS6_F	UBOCC31005	CCTATGGCCATATTCAAAAGCA
	TerS6_R		CCCAACTGACTACCAAGATTTG
terS7	TerS7_F	Vinitor 162	ATCAAGCTGGTAAAGACAATGG
	TerS7_R		CTGTTCCATGACTTCGATTTGT
doc	Doc_F	IOEB0608	TAACGGCCTTGATAGCGTTT
	Doc_R		TTGAGGCATCCTTCCTTCAG

Table S3. List of spacers in the four CRISPR loci identified in the *Oenococcus* genus.

Type II CRISPR in *O. kitaharae* DSM 17330[T]

K1	TGCACCATGTGCCATATCATCGGCATTGCC
K2	ATAATCACCCAGCTAGGATTTGTACATGGC
K3	AAGAATTCTGTCAAATGCTAAAGACAGATA
K4	CACCACTTTTTACAAGCTCTGTTCTAATGG
K5	GCTATATCGGCCAAACACAAGACCAACAAG
K6	TTGCTTCTGTCGGGAATGCCGTGACTCAAG
K7	AAAGTTTGGTCATTTTTTTACCTCAATCAT
K8	TTCAAACAAAGCAATTGGTCGAAACATTGG
K9	TGATACGATTGACTTTCAATATCACCAAAC
K10	TAATTGATACAAACGGCGAAGTGTTAAGCA
K11	ACTGTGATTTGCCTGTACGTGTGAAAAACG
K12	TGGAACGGTGCTGAGAACCAATGGTTATGC
K13	GTGATATTTGGATTAGGTGAAACAGAAACG
K14	ACAACCTCAACTATTTGAGTGTTGGCAAGC
K15	GCAACGAATAGGCCGTACCGACCTATTCGT
K16	AAACGGATATTATCAATTTGAGGTAATCGC
K17	TTCAGTGGCTCATAACCCATTGGCATTTGT
K18	ATGATTTGAAAACAATCTTGCAGATAAAAG
K19	AACGCATTTGCTGGAATTCGGACACGCCAC
K20	AGTACAATACGGATGAAATCAGCATGTCGA
K21	ATTCATGGGCTTAGGCACACACATGCGAGT
K22	ACAATCGTGTGCTTATCGCCACGCCATGGC
K23	GGTTGGATGACCATAATTTGCTGGGCTTTG
K24	AATCCTTGAGCTAAATTACCGTTAATCGTA
K25	TTTATTACTCCTTTCAAGCTGACTGTTGGG
K26	TAGCTGCACTGGCAGAAACACGGGCATTAA
K27	AAAATATCGTCAATACACAATCAGAAGGTT
K28	TTTCTGTCATCTCGATCCTTTCTAAATTAA
K29	AGAATGCGTTGTTTATCAAGGCTCAAAGTG
K30	AGTTATCAAATCAAAATCGAGAGTATTAAC
K31	GAGACCACTGGCTCTATGATGGAAAAGTAG
K32	CTACTTCTATTGAAGTAGTACTACCAAGCA
K33	AAGTAAATGGTACAAAAGCTCTCACAACGC
K34	TCTTGGCCGGTATCGGAAAGGGCATTGCTA
K35	AATTGCTTAGAGAGCGATTTTTCAAACAAT
K36	TACCACATTCTTCAACAGACTATCAGTCCT
K37	AACTAATTTATTCCTTTTAAGGAACATATT

K38	GCGTATATGCTCAAACCGGAGTTCATAGAA
K39	ATAGCGACGTTGGCTAAACAACCAAATGGG
K40	TTTATCAGGTTTCTCTAAATAGTTCTGCCA
K41	TACAAAGCCGTTGGTAATTTATCCGGTTCA
K42	TTCACTTCCTCTTTTCTGAGCCCAGAAAAT
K43	AAGCGGTGGTTCATGAAGCTCGCCAAAATT
K44	CTGATAAACGACGGAATTTTGTCCCTTGAC
K45	ATTTCAAAGCAATTTCGCAAAAAGATCAAA
K46	GAAAGCAAATCTTATCTCGTGACGCAGGTG
K47	TAGAATCAATCCCCTGTGCTTCGTCTCTTG
K48	GAAGGCTACATCAAGACTGGTGTCGATCAT
K49	CCGTTGGCATGCAACCAGATGCCAACAACC
K50	AACCGTTCAGTCCATAAACGCCGGCGCTAT
K51	CGGCAAAGCGGAATAATCTTGTCACCTTCG
**K52**	TACAGCTTACAAAATACATAAAAGTATGCA= K55
K53	TTATTTAAAAGCCGGTCAACATCCTTTTAA
K54	CATTTTTATCTGTAATGTCACTAAAAATAT
**K55**	TACAGCTTACAAAATACATAAAAGTATGCA= K52
K56	TTTCCCGTGCGTGGGAAAACCCTGATTTAA
K57	CATGCTGGTCTTGTATGACACCAAACATGA

Type II CRISPR in *O.* ciserae UCMA 15228^{T}

S2.1	AGAAATTTTGGAATTTATATAATCACAAAG
**S2.2**	GCGGTTCAAAGACTGCGATCAGTTTAATTC = S1.3
**S2.3**	GCGGTTCAAAGACTGCGATCAGTTTAATTC = S1.4
S2.4	GGACTGCGAAGACAATCGGCAAGAAATCAC
S2.5	GTATCGGTGTCTTCATCAAAGTCTGGTTCG
**S2.6**	GCGGTCTAAGTCGTATTGAAGCCAGTGGCA = S1.7
**S2.7**	GCGGTCTAAGTCGTATTGAAGCCAGTGGCA = S1.6
S2.8	TAATAATACCAGCTGCGGGAATATTTAGTT
S1.9	GCGATTGATGCGCTCCACCTCGCTGGTGTT
S2.10	AGTATCAGCGAGCTTATACCGCTCTGTCAT
S2.11	ATCAATGAAAACATGAAAACCCCTTACACT
S2.12	CCATTCTGTGACTCCTTTTTAGCTTTTCTTG
S2.13	GAACTCGGACTTGCTAAATCTTTTTTGTAA
S2.14	TGGACGTCGAAAATGCACGCTGGCGTATCA
S2.15	ATGGTTACGATAACTTGTTGGCTGCCTTAG
S2.16	GAATATCCGAGCTTCTCTATCACACTTGCG
S2.17	ATTTCAAACGAAGCCATCGCAGAATTGACA

S2.18	GAGGGAGCTATTGGTAACTTGCAAGCCAAC	
S2.19	ATGGAATCAACGAAGAGCAAGCCCAAGAG	
S2.20	TTCATATCATCTATTAAATCACTATATAAA	
S2.21	TATCTGACGCTGTTGGTATACAAGTCACAA	
S2.22	CCAACTAATTGCTTTATTGATATTAGCAAT	
S2.23	TGAAAGACGACTACGTTTACGGACAATTGG	

Type I CRISPR in *O. ciserae* UCMA 15228 ^{T}

S1.1	CTTATAACCAATGACAAAGAAAGCAATGTTAT	
S1.2	TTATTTTTCAAGGCAAAGATGATGGCCGTTGT	
S1.3	GCACCGAGTTCGTTCGCCGTTTCGTTTATTTT	
S1.4	TTTTTGATTTCGTCATCGATATTTTTAATTGG	
S1.5	CGATTGACATTGTCGTTCGGCATGATCCGACT	
S1.6	TGCTTTTCAAACCGTTTACCCAATTTTCTACC	
S1.7	TGTGCTGCTCATGGCTTTCGTCATGACTTTACC	S2.17
**S1.8**	TTCATCAAACAGCCCTAATTTTCCATTTGTCC	S2.18
S1.9	AATCGTAATATGCGTCGAGCAATCAACTTGCA	
S1.10	TTTTAAGTATGCCAGCAGGAACATTGGAGCGAC	
S1.11	TTATAAGCACCCTCTTCATTATGCAGGCGGGC	
S1.12	CAAAATGCTTCTCGAGGCGTGAATGATGGAAT	
S1.13	ACCCTTTCATGTTTATCTTAACTTTCGGCCAC	
S1.14	TGGCTATTTTGGCATCTGCCTGGGCTTTTTCT	
S1.15	CCGCTGATTTACCAGATGATCTAAGTGATAAA	
S1.16	ACAACGTTGATGAAAAATACGTTGATTGGACT	
S1.17	TGTGCTGCTCATGGCTTCGTCATGACTTTACC	S2.7
**S1.18**	TTCATCAAACAGCCCTAATTTTCCATTTGTCC	S2.8
S1.19	CTCTTTTTGCGCTTGCGGTTGACCCTCCCACC	
S1.20	GCAGAGGTTAAGGAAACTTTGAAGTTATCATG	
S1.21	TACATAAATATCAGTATTGCAAGTTTAAACGT	
S1.22	TTCCATCAGATGAAGTTTTGCCGTTCTAAGCG	
S1.23	TACACGACAATGACCAGAACTATGAATTATTG	
S1.24	TGGAACGGTATTATCGTTGTGGCCACGGCCGT	
S1.25	TCGCCTTTTAAGGTTTTAACGGCGTACTTGCT	
S1.26	TGGTTGATGCAGCCAAAAATCTGCGCTTAAGC	
S1.27	ATTTCGGCAATATTTTCAATTCATGAATTTTC	
S1.28	CCACCTTTGACAGCGGTTACTGAAGACTGGCT	
S1.29	AAGAAAGCCTAACCGAAAAATACCAAAATTAT	
S1.30	AATGTGCTTGATTCAAGCTATGGCAATGACTC	
S1.31	TGATTTGCAAATCATCGAGGAGGAAAAAAATG	
S1.32	TTTCATTTAAATATGCATGCTGATGATGACGG	

S1.33	ACGGTCACATTATCGCCAACATACGATCAGGA	
S1.34	TCCCATGGGGTTCAGGCCTGAAAAGTGTTGCT	
S1.35	CTTTTCCTGCCGTTTCTGCCATTGCTTGCTCC	
S1.36	CCCACCAGCTGTCTGGCATTGAGATAGTTCGT	
S1.37	GTAGTCATGGACAATTCAGCACAGATTCAATC	
S1.38	GATTCAAAAAATTAGTTTTAATCGTCGTATCA	
S1.39	GTCATCTAACAAGCTTACAATTTGGTCCCTTA	
S1.40	TTAATGGTTACTCACAGGGCGAAGCTACATTT	
S1.41	TTGCACGTTGGCTATGAATAGCACATATAGCC	
S1.42	CATCATCAGGATCTTCCCGTTCAATTTTGGTT	
S1.43	ATTAAAACGTAAATATAACATTCTAAAAGCAG	
S1.44	ATGGCCTAACCATACGAATGGGTGGGAAGCCC	
S1.45	GAATTCAGGTTATGAACCAGAATCTTTGCCAT	
S1.46	GATCCGTTGCATGATTCTTATGCAGCTGCCTT	
S1.47	CAAGTTTGATAAGTTAGTTATGTTTAGTGGCA	
S1.48	CAGGAATTTTCCAGAGAACCAGACCTATACCA	
**S1.49**	ACGGTACGATTCCGCTCTATCAGTCAACGATT	S1.56 & S1.62
**S1.50**	ATCAGGTGGGCGTTTCTGACGCTATGAACATT	S1.63
**S1.51**	TGACATGCACTGGTCGGAGATGTTTTTTGATG	S1.64
**S1.52**	AAATTCACAGCACCGACCATGTGCGGTCTGAT	S1.65
**S1.53**	TCAGCTTCTCGAGACAAAGGTGCAATCTGATA	S1.66
**S1.54**	AAAAATTAATAATTTTTGCGCCAGATTCAGCA	S1.67
**S1.55**	ATTGAGACGGCCCTACATAAAATTTTGAAGAG	S1.61
**S1.56**	ACGGTACGATTCCGCTCTATCAGTCAACGATT	S1.49 & S1.62
S1.57	ATCGTGTTAATGAAGCCTTATCAGAAGTTACA	
S1.58	TCAGCTTTGTGATGGCTAACAAGGACTGGCTT	
S1.59	TAGCTCACGACGAAGGCTTGACAGAGTTTGCC	
S1.60	TTGCAAGAGGCTTTGGGACAATATTTTGATAA	
**S1.61**	ATTGAGACGGCCCTACATAAAATTTTGAAGAG	S1.55
**S1.62**	ACGGTACGATTCCGCTCTATCAGTCAACGATT	S1.56 & S1.49
**S1.63**	ATCAGGTGGGCGTTTCTGACGCTATGAACATT	S1.50
**S1.64**	TGACATGCACTGGTCGGAGATGTTTTTTGATG	S1.51
**S1.65**	AAATTCACAGCACCGACCATGTGCGGTCTGAT	S1.52
**S1.66**	TCAGCTTCTCGAGACAAAGGTGCAATCTGATA	S1.53
**S1.67**	AAAAATTAATAATTTTTGCGCCAGATTCAGCA	S1.54
S1.68	ATTACGTCAATGATTTCATTGATTGAAGCATT	
S1.69	TAGCAGCGGCACTAAAAATAGATCCTTGGGAG	
S1.70	GGTTACTTCAAGGAACAGCTTTTGTGCTGCTA	
S1.71	CCTCAGCTTTTGCTTTCCGCTGGGCTGCTTCG	
S1.72	CGTGAGCTGCTGCAGCAAATGTACGGAAAATC	
S1.73	AAATGACCAAAAAACTTTTTGCGAATCTGGGC	
S1.74	TGCAATCTTGGAGCAATCAGCAGGTAAAGGGT	

S1.75	CCGTTAGTTTGACTAATTGGGACTTTAGTTGA
S1.76	CTACAATTAGATTCTATTTCTAGTTATCAAAC
S1.77	TCCCATGGGGAATAAATCTGAAAAGTGTTGCT
S1.78	AACAACCTAACAGAATTAATCGGTGGCCTGTT
S1.79	CTAATTTTATTTGTCATTTTTGTACCTCTTTA
S1.80	AAGCTAGACGAAACACCACTATTGACACTGGC
S1.81	TTGTCTCGATGAATACGGTCATATTCAGTTTG
S1.82	TATCAGAGTCGACGCTCCCGCTGTAACCGCCG
S1.83	TGTGCCTCAAGCCATGAAAAGTTATTTGTTTA
S1.84	GGTTTCCTGTCCAGTTTCAAAGAAAAACTGTC
S1.85	TCAGCCCAGTTCGTCGAAATGAACTGCGAAAT
S1.86	ATTTATTAGGACGAGGGACTCCGATGGTGAAC
S1.87	ACCATCGTTTGATAAGTCGAGCTCTCAACTTT
S1.88	GACTAGCAACAAATCGACAACAAAAGATCCTT
S1.89	TTTCTAGTTCTGATAACTAAGTCAGAATTACC
S1.90	GAACGGTTAAATTAACACTTAATGGAGTAGTA
S1.91	TTCAACAAGACGCTGACAATGCCAAACTAGAT
S1.92	TTCAAAAAGGGAACGTTAACCCTGCCACAGAT
S1.93	AAGCACGTATTTCCGGTTCGCCAGCCATCACG
S1.94	TTTGTTTTGGGCTTGTCTATGGGCGTACTGAT
S1.95	CTCAGTCATTTCGACCGTGTATCGCTTGTTAT
S1.96	AGCCCTATTTTATCTTTTCCCCGTCAATATAT
S1.97	TTTTTTATCAGTCAGCCCAAACAATTCCCGAT
S1.98	CAAGAAGTTCCACTAATCACTGTCGTCGCAGC
S1.99	TTGATTTGCTGCAATGGCTTGTGTTGGCTTAG
S1.100	GTTGGCGTGGCAATACTTGCCGTGCCGGTACC
S1.101	ATCCTGTTATGTGATTCATTGTACAGCACACA
S1.102	CAAAGCCTTGGGCCATAAAACCCAAGCCTTCCC

Type I CRISPR in *O. alcoholitolerans* UFRJ-M7.2.18 (chromosomal DNA)

A1	CCCATTTTAATCATTTGCAGATAATCGACT
A2	CTAACAGCTAAACAGAACTCTATTGCTAGC
A3	CAAAACGGTAAAAGTAACGCCAAAGGGTCA
A4	CGTTAGATAAAATCCATCAGCAGGAGAAGT
A5	CAAAGTCAGACAACCGTTGTATATATTGGC
A6	CATCAATTCATTTAATTTATCATAATCAAT
A7	CAACAGTTTCTATCGTACTCAAAGGAACCC
A8	CTTGAATTGCCAAAAGAAAACTGGGCGTTA
A9	CTATGGCGATAAATTCAACTGATTCAAATG
A10	CTCTAAATATGGTCAACTATGATGCATATG
A11	CACCAACTGCCAGCTGTTCCAGAAGTATAA

A12 CGTCACATCGACAAATATAGCTAACGGGCT

A13 CCTCGGACTACCTCCACAATGGTGTTGACC

A14 CTTATTAAAATGATATATACTTGTTCTCGTT

A15 CTGAGAAGTTTGGCGAAATCAAACCCTCTG

Miscallenous in *O. alcoholitolerans* UFRJ-M7.2.18 (plasmid DNA)

Plasmid II

AACGGACGCTAATCGGACGCTAATTTAAATAACGAAAGTAGCAAAA

Plasmid III

TGGGCGGTCTTGTCAACGGACTTATGCG

Table S4. Rapid sub-typing scheme amongst oenophages showing 27 distinct patterns.

Prophages	Int A-F	LM 1-6	Rep 1-5	TerS 1-7	TMP 1-7	Doc	Pattern
IOEB0205	A	1	1	1	1	+	1
OE33PA6		1	1	2	1	-	2
IOEB0608		1*	1*	3*	3*	+	3
IOEB1491		2	1	2*	1	-	4
IOEB0501#		2	2*	1	1	-	5
L26-1		2*	2	1*	2*	-	6
AWRI B422		2	2	2	1	+	7
CRBO14223#		2	2	2	1	-	8
S161#		2	2	2	2	-	9
IOEB0502		3*	1	4*	4	-	10
CRBO14203		4*	1	1	1	+	11
AWRIB508#		4	2	2	1	-	12
AWRIB847#	B	1	2	1	2	-	13
CRBO14210		1	2	3	3	-	14
AWRI B424		1	2	3	3	-	
CRBO14221		4	1	1	1	-	15
AWRIB133		4	2	3	1	-	16
CRBO11105#		5	2	1	1	-	17
AWRI B202#		5	2	1	1	-	
OE33PA		5	2	1	1	-	
AWRIB150#		5	2	1	1	-	
IOEBB10#		5*	2	2	1*	-	18
AWRIB127*	C	1	1	1	1	-	19
AWRIB304		1	1	2	2	-	20
S28		1	1	3	4*	-	21
IOEB9805	D	6*	3*	5*	5*	-	22
AWRIB418		6	4	5	5	-	23
CRBO1384	E	1	1	4	4	+	24
AWRI B663		4	3	5	5	-	25
UBOCC31005	F	1	4*	6*	7*	+	26
Vinitor (162, 27, 28)		-	5*	7*	6*	-	27

Phages belonging to clusters I and II are in orange and blue, respectively; #, phages from cluster I.1 which contain several protospacers; *representative LM, Rep, TerS, TMP and Doc proteins (see Figure 2).
